# Gelsolin pathogenic Gly167Arg mutation promotes domain-swap dimerization of the protein

**DOI:** 10.1093/hmg/ddx383

**Published:** 2017-10-23

**Authors:** Francesco Bonì, Mario Milani, Alberto Barbiroli, Luisa Diomede, Eloise Mastrangelo, Matteo de Rosa

**Affiliations:** 1 CNR Istituto di Biofisica, c/o Dipartimento di Bioscienze, Università degli Studi di Milano, 20133 Milan, Italy; 2 Dipartimento di Scienze per gli Alimenti, la Nutrizione e l'Ambiente, Università degli Studi di Milano, 20133 Milan, Italy; 3 Dipartimento di Biochimica e Farmacologia Molecolare, IRCCS – Istituto di Ricerche Farmacologiche ‘Mario Negri’, 20156 Milan, Italy

## Abstract

AGel amyloidosis is a genetic degenerative disease characterized by the deposition of insoluble gelsolin protein aggregates in different tissues. Until recently, this disease was associated with two mutations of a single residue (Asp187 to Asn/Tyr) in the second domain of the protein. The general opinion is that pathogenic variants are not *per se* amyloidogenic but rather that the mutations trigger an aberrant proteolytic cascade, which results in the production of aggregation prone fragments. Here, we report the crystal structure of the second domain of gelsolin carrying the recently identified Gly167Arg mutation. This mutant dimerizes through a three-dimensional domain swapping mechanism, forming a tight but flexible assembly, which retains the structural topology of the monomer. To date, such dramatic conformational changes of this type have not been observed. Structural and biophysical characterizations reveal that the Gly167Arg mutation alone is responsible for the monomer to dimer transition and that, even in the context of the full-length protein, the pathogenic variant is prone to form dimers. These data suggest that, in addition to the well-known proteolytic-dependent mechanism, an alternative oligomerization pathway may participate in gelsolin misfolding and aggregation. We propose to integrate this alternative pathway into the current model of the disease that may also be relevant for other types of AGel amyloidosis, and other related diseases with similar underlying pathological mechanisms.

## Introduction

Gelsolin is the prototype of a large superfamily of proteins, responsible for the assembly, disassembly and scavenging of actin filaments by means of its severing and capping activities (1,2). Gelsolin is organized into six homologous domains (G1–G6), sharing the same gelsolin-like fold (3). Each of these modules hosts at least one calcium-binding site and the presence of calcium induces both subtle local and large global conformational changes (4–7). Namely, actin-free gelsolin exists in two functional states: in the absence of calcium, the protein adopts a compact conformation unable to bind actin (inactive form); at higher calcium concentrations, the gelsolin structure unwinds leaving the actin binding surfaces exposed (active form) (8,9). The active conformation is highly dynamic as most of the inter-domain contacts are lost and the domains are linked only by flexible stretches of the polypeptide chain (9). For this reason, no high-resolution structure of calcium-activated gelsolin has been reported so far and structural biologists have to mainly rely on the study of isolated domains to gain insight into gelsolin physiopathology (3,4,6). In each domain, calcium has a structural role and its binding leads to the stabilization of the gelsolin-like fold and consequently, to decreased conformational flexibility, which is somehow in contrast to the effect of calcium on the full-length protein (5,6). 

Owing to its pivotal physiological activities and its ubiquitous nature, gelsolin, both in the intracellular and extracellular form, plays a major role in a plethora of physiological processes, such as cell motility and cell division, organelle trafficking and muscle contraction (10). Increasing evidence suggests that intracellular gelsolin exerts a central role in cell metabolism and signaling through an actin-independent mechanism (11). Alteration in gelsolin expression levels or deregulation of its activities have been observed in several pathological conditions, including cardiovascular diseases, muscle necrosis, acute respiratory distress syndrome and in particular, cancer (12). However, a clear relationship between the gelsolin levels and its effect in protecting or inducing a specific pathological condition remains to be elucidated. Conversely, the presence of mutations in plasma gelsolin is known to be directly related to a rare form of amyloidosis, hereafter referred as AGel amyloidosis (AGel, OMIM reference number 105120). 

Amyloidosis-related diseases are degenerative in nature, with pathogenicity stemming from incorrect protein folding, which leads to the deposition of insoluble protein aggregates (amyloids) in various organs and tissues. More than 20 amyloidogenic diseases, including central and systemic forms, have been identified. They can be either hereditary, owing to mutation(s) or deletion(s) in the etiological protein, or sporadic, i.e. associated with external factors. 

The first genetic form of AGel, which until recently was the only one described, was originally known as familial amyloidosis, Meretoja’ syndrome or Finnish-type, owing to the clinician who first described the disease and the country where the pathology is endemic, respectively (13). Finnish AGel is caused by the substitution of Asp187 to either Asn or Tyr (numbering according to the mature plasma protein or G640 to A or T in the mRNA sequence) (14,15). Systemic amyloidosis is clinically characterized by the prototypical symptomatic triad associated with the deposition of gelsolin fibrillar tangles in the eyes, skin, peripheral and central nerves. Since its discovery in the late 60s, AGel cases have been described in many other countries [some recent examples in (16–18)], in kindred lacking Finnish ancestors, suggesting that this disease, owing to its neglected state and complex and variable clinical picture, has often been un/misdiagnosed.

Two novel pathological variants of gelsolin have recently been described to be associated with renal amyloidosis containing the following mutations: Asn184 to Lys and Gly167 to Arg (C633 to A and G580 to A, respectively) (19–21). In addition, a sporadic form of AGel with marked wild-type (wt) gelsolin deposits surrounding a sellar glioma of the hypophysis has also recently been discovered (22). This finding is particularly relevant since, in several clinical reports of pituicytoma-associated amyloidosis (23–25), it is the first one in which the etiological agent, i.e. the main constituent of the aggregates, was identified. Therefore, AGel can be classified into three different forms according to the nature of the protein, mutants or wt, in addition to the organ(s) involved in fibril deposition: (i) systemic (or Finnish-type), (ii) kidney localized and (iii) sporadic.

All AGel types share the lack of apt pharmacological therapies that cure the disease, targeting the source of toxicity, rather than only acting as palliative, symptomatic treatments. However, the use of nanobodies raised against mutated gelsolin recently showed great potential as a novel strategy against AGel, both *in vitro* and *in vivo* exploiting adeno-associated viruses (26–28). Dissection of the molecular bases that lead to wt and mutant gelsolin misfolding, and knowledge of the mechanisms underlying each AGel form, are crucial to identify pharmacologically relevant targets and to develop effective therapeutic strategies.

With the exception of our recent description of the underlying molecular bases of Asn184Lys amyloidosis (29), little is known about the pathological mechanisms that lead to renal or sporadic forms. On the contrary, the amyloidogenic pathway of the systemic Asp187Asn/Tyr variants is relatively well understood. Asp187 is present in a cluster of residues that chelate calcium in the G2 domain (6,30), and its substitution compromises calcium binding and leads to overall domain destabilization (31). Such increased flexibility makes the protein susceptible to aberrant proteolysis, triggered by endogenous furin in the Golgi (32). The major product of furin activity, the C68 fragment, becomes a substrate of other enzymes and the proteolytic cascade eventually leads to the production of two aggregation prone peptides (5 and 8 kDa). Therefore, in Finnish amyloidosis only the exported isoform of the protein is responsible for the disease and the full-length mutant protein is not *per se* amyloidogenic, only the short fragments are found in fibrillar tangles (33,34). These findings however, are in contrast with laser dissection studies, followed by mass spectrometry analysis of Gly167Arg deposits that identified gelsolin fragments that do not correspond to the canonical 5 and 8 kDa peptides (19), suggesting that either the full length protein aggregates or this mutant is characterized by a different proteolytic pattern.

The effect of Gly167Arg substitution in the G2 domain of gelsolin on the amyloidogenic pathway is investigated here. We report the first crystal structure of this protein variant and show that the Gly167Arg mutation promotes gelsolin dimerization *via* a peculiar 3D domain swap mechanism. Our results challenge the current paradigm on gelsolin aggregation and propose an alternative, proteolysis-independent pathway for the deposition of the Gly167Arg variant. The domain swap versus proteolysis mechanism may explain the observed differences in fibril localization and the clinical picture induced by different mutations. Owing to the generality of the proposed mechanism, our findings may be relevant to the other AGel forms and to other similar diseases, whose pathological mechanisms are still under investigation.

## Results

### The structure of the gelsolin domain 2 Gly167Arg mutant

#### Domain swap mechanism

**Figure 1.**
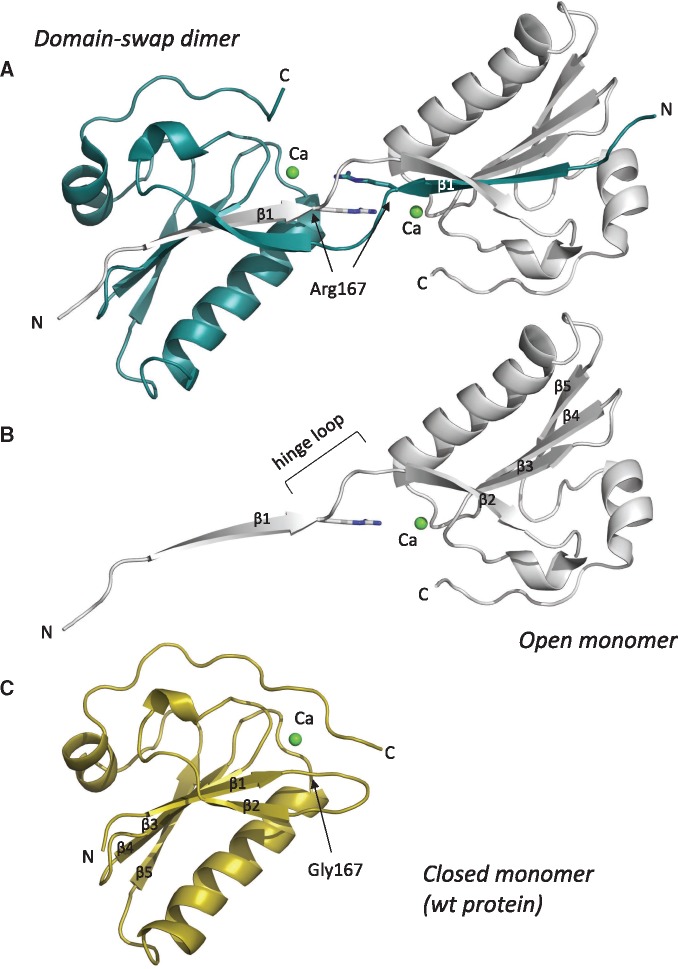
Gelsolin Gly167Arg domain 2 structure. Cartoon representation of (**A**) the domain-swapped dimer, (**B**) the open monomer as extrapolated from the previous assembly and (**C**) the closed monomer from the wt structure (PDB id 1KCQ). All structures are viewed in approximately the same orientation; N- and C-termini, the mutation site, the bound calcium ion and the β-strands are labeled. The hinge loop, which is the region of the protein that links the swapped domains, is also indicated.

**Figure 2.**
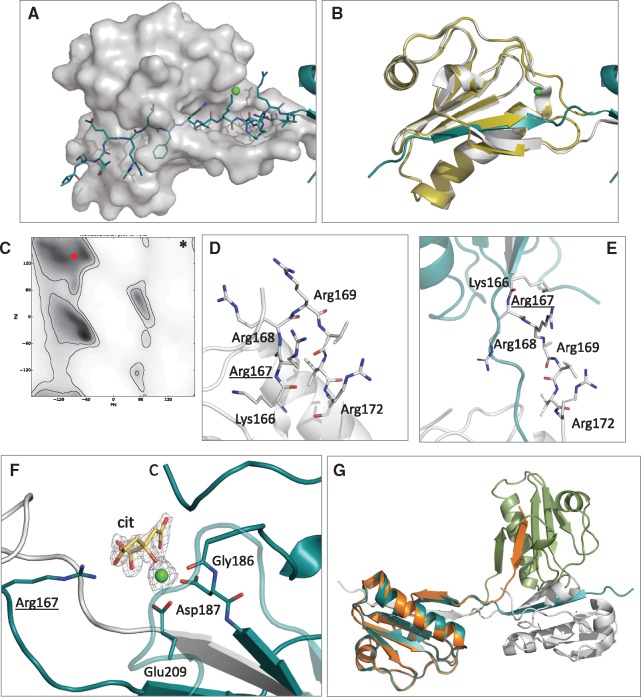
Details and characteristics of the domain-swap dimer. (**A**) Interactions between Chain A (transparent surface) and swapped strand β1 of Chain B (cyan sticks). (**B**) Superposition of wt monomer (yellow cartoons) with chain A of the Gly167Arg dimer (white cartoons). (**C**) Ramachandran plot for arginines (gray gradient indicates most favorable angles), the red asterisk shows the position of Arg167 in the dimer (from the reported structure) while the black symbol the corresponding Gly167 in the wt monomer. (**D** and **E**) Zoom view of the hinge loop in a model of the mutant monomer and in the crystal structure of the domain-swapped dimer. Positively charged residues hosted by the segment are shown as sticks. (**F**) The calcium-binding site in the Gly167Arg dimer showing the calcium ion (green sphere), chelating residues (sticks) and Arg167 that forms a H-bond with the citrate molecule (cit., in yellow). All residues are labeled. 2Fo-Fc electron density, contoured at 1.5σ, for calcium and citrate is also visible as a gray grid. (**G**) Superposition of chain A of the dimer (cyan and white cartoon) with chain A of the lower resolution monoclinic structure (green and orange), to highlight differences in the relative orientation of the monomers in the two crystals.

In a previous study, we successfully used a combination of biophysical and biochemical approaches to understand the role of the Asn184Lys mutation in the amyloidogenicity of gelsolin (29). A similar approach was here applied to dissect the molecular bases of the pathogenicity of the other variant responsible for gelsolin-related renal amyloidosis. To this end, we used the G2 domain (amino acids 151–266) carrying the Gly167Arg mutation, for extensive crystallization trials. Crystals readily grew in 0.2 M ammonium acetate, 0.1 M sodium citrate, pH 5.6, 30% PEG 4000 and the structure was solved by molecular replacement using the Asn184Lys variant as a search model (see Table 1 for data collection and refinement statistics). Contrary to the previously published gelsolin G2 crystal structures, two molecules were present in the asymmetric unit and during refinement we observed a discontinuity in the electron density corresponding to a loop connecting β-strand β1 to β2 (hereafter named as the hinge loop) (Supplementary Material, Fig. S1). Indeed, such discontinuity was owing to an unusual conformation of the crystallographic dimer, with the N-terminal β1 strand of each monomer contributed from the other polypeptide chain (Fig. 1). This rare mode of association is known as 3D domain swapping [reviewed in (35,36)], whereby secondary structure elements or a structural domain of one protein is replaced by the same element donated from another protein. Although domain swapping has already been associated with both physiological and pathological processes, our structure is the first example of a gelsolin mutant that adopts such a conformation. The dimerization interface between the two monomers mainly involves the swapped β1 strand, comprising residues 158–166, covering a total contact area of 2375 Å^2^ (Fig. 2A). In addition, the two stretched hinge loops interact in a pseudo β-antiparallel fashion, contributing several polar interactions to the assembly (Fig. 2A and E and Table 2). All inter-domain interactions (more than forty H-bonds) belong to the interface between the open monomers [*O*-interface according to Eisenberg nomenclature (36)] and no contact occurs between the closed subunits (*C*-interface).

**Table 1.** Data collection and refinement statistics

Data collection
Beamline	ID30B (ESRF)
Wavelength (Å)	1.01
Space group	C 1 2 1
Cell dimensions	
*a*, *b*, *c* (Å)	106.3, 44.4, 58.0
*α*, *β*, *γ* (°)	90.0, 110.1, 90.0
Unique reflections	26, 967
Resolution range (Å)	45.40–1.70(1.74–1.70)^a^
*I*/*σ*(*I*)	9.82 (1.91)^a^
*R*-meas (%)	12.4 (80.2)^a^
Completeness (%)	95.5 (98.5)^a^
Multiplicity	3.5 (3.5)^a^

Refinement

Resolution range (Å)	45.39–1.70(1.77–1.70)^a^
*R*_work_/*R*_free_ (%)^b^	19.9/24.2(34.9/34.4)^a^
r.m.s.d.	
Bonds (Å)	0.011
Angles (°)	1.066
Ramachandran plot	
In preferred regions (%)	96.5
In allowed regions (%)	3.5
B-factors (Å^2^)^c^	19.4

Statistics of the domain swapped dimer of the Gly167Arg G2 gelsolin mutant. Model and structure factors have been deposited in the protein data bank (http://www.rcsb.org/pdb/home/home.do) under accession code 5O2Z.

a Values in parentheses refer to the highest resolution shells.

b *R*_work_ = Σ_*hkl*_‖*F*_o_|−|*F*_c_‖/Σ_*hkl*_|*F*_o_| for all data, except 5%, which were used for calculation of the *R*_free_.

c Average temperature factors for the overall structure.

**Table 2.** Characteristics of the intertwined assembly

No. of swapped residues^a^	9 (158–166)
No. of residues in the hinge loop^a^	5 (167–171)
No. of intermolecular H-bonds (salt bridges)^b^	41 (13)
Buried interaction area (Å^2^)^a^^,c^	2375
Total interaction surface (Å^2^)^a^^,b^	2987
r.m.s.d. wt monomer versus G167 dimer (Chain A) Å (no. of Cα atoms)	0.4 (79)

Physico-chemical parameters of the domain swapped dimer. The table lists parameters similar to those cited in (36) to compare our structural features with those of other domain-swap proteins previously characterized.

a Values are intended as ‘per subunit’.

b Computed using the PISA software.

c Calculated by subtracting the solvent accessible surface area of the closed monomer from that of the open monomer.

All attempts to obtain the structure of the mutant in the monomeric form failed, therefore we compared the Gly167Arg dimer with the wt G2 structure. If the hinge loop (residues 168–172) and the C-terminal region of the domain (residues 258–265, not visible in the mutant structure owing to its flexibility) are not included in the superposition, the two structures are highly similar with root mean square deviation (r.m.s.d.) of 0.40 Å (over 79 Cα atoms) (Fig. 2B and Table 2).

Analysis of the Gly167Arg mutant provides clear hints about the structural bases of the dimerization process. In both wt, and to a great extent in Asn184Lys G2, the hinge loop exhibits some flexibility (6,29). The loop also displays some *turn* characteristics, as the residue 166 (n) forms an intramolecular H-bond with residue 172 (n + 6), and in position n + 1 the loop hosts Gly167, which sits in a peculiar region of the Ramachandran plot, specifically accessible only to glycine and definitely unfavorable for other residues (Fig. 2C). Contrary to this, the stretched conformation of the hinge loop adopted in the Gly167Arg dimer better accommodates the bulky residue, eliminating the torsional strain. Furthermore, the mutation adds a basic amino acid (Arg) to the hinge loop, which already hosts three arginines and one lysine, attributing five positive charges to a short stretch of seven amino acids (Fig. 2C–E). This is an extremely high concentration of charges that extend over a limited area, which necessarily leads to electrostatic repulsion. Therefore, the Gly167Arg mutant triggers the conformational change to release both the torsional strain and the repulsion among the basic residues.

In addition to the described structure, we collected a second low resolution dataset for the Gly167Arg mutant in a different space group (P2_1_) and the resulting 3D model was partially refined to 2.6 Å resolution. Also in this crystal, the mutant forms a dimer stabilized by the same domain swapping mechanism. The relative orientation of the two subunits differs between the two structures, both in terms of the angle between the monomers and rotation along the symmetry axis (Fig. 2G). This evidence suggests that the mutual orientation of the two monomers in the dimeric assembly is mostly dictated by crystal packing rather than by inter-domain interactions, demonstrating the intrinsic flexibility of the hinge loop.

#### Calcium-binding site

Although no crystal structure of Asp187Asn/Tyr variants is available, calcium-binding impairment was reported as the primary cause of their amyloidogenicity. Solution and computational studies on these mutants showed that in the absence of calcium, the C-terminal tail of the protein is highly dynamic with a tendency to expose the empty calcium-binding cavity (6). In one of the two structures of the Asn184Lys mutant we observed similar behavior, although the calcium ion was still firmly bound (29). Similarly, in the Gly167Arg dimer structure, we could only model up to residue 257 and 258 in chains A and B, respectively. The remaining 7/8-residue segment was too flexible to be modeled, suggesting that destabilization of the C-terminus of the G2 domain is a feature common to all gelsolin pathogenic variants described to date (Figs 1 and 2F). Dimerization itself is partially responsible for this behavior as the C-terminus would clash against the hinge loop in the conformation observed for the wt protein. Surprisingly, neither dimerization nor destabilization of the C-terminal segment impair the ability of the mutant to bind calcium. The electron density for calcium is clearly visible and its occupancy was refined at 100%. In the other G2 structures, calcium is hexacoordinated by Gly186, Asp187, Glu209 and Asp259, but the last residue is disordered in the Gly167Arg mutant and, in its place, we observed a citrate molecule in both subunits (Fig. 2F). Citrate was derived from the crystallization condition (0.1 M concentration) and was H-bonded to residues 186, 204, 206 and the mutated 167 residue. Citrate binding does not seem to be the cause, but a consequence, of the opening of the C-terminus, since in the Asn184Lys mutant, in the absence of citrate, water molecules replace Asp259.

#### Molecular dynamics simulations

As the structure of monomeric Gly167Arg G2 is unavailable, we performed two molecular dynamics (MD) simulations (80 ns) using the coordinates of the *in silico* mutant structure of the wt G2 domain (pdb-id 1KCQ) in order to analyze the impact of the mutation on protein dynamics, either in the presence or absence of calcium.

When G2 is bound to the calcium ion, the Cα r.m.s.d. value with respect to the initial model is promptly stabilized at ∼1 Å (Supplementary Material, Fig. S2A), and is maintained throughout the simulations, suggesting that structure stability is kept in the mutant. During the simulations, the main chain of Arg167 is constrained in an unfavorable region of the Ramachandran plot with a Phi of ∼120 ± 25° and a Psi that alternates around +150° and −150° (Supplementary Material, Fig. S2B). Arg167 is maintained in this unfavorable conformation by a H-bond formed between its main chain nitrogen and the side chain of Glu209, which is in turn bound to the calcium ion. Therefore, from the MD simulations, even in the presence of the geometric strain induced by the Gly to Arg mutation, the role of calcium in maintaining the native-like conformation of the hinge loop clearly emerges.

On the contrary, simulations performed in the absence of calcium, showed that the increased motility of the hinge loop relieves the torsional strain imposed on Arg167. This higher conformational freedom is also reflected by the overall Cα r.m.s.d. with respect to the initial structure that continuously increases during the simulation time (Supplementary Material, Fig. S2A). Interestingly, without calcium the secondary structure elements around the hinge loop start to lose their integrity (Supplementary Material, Fig. S2C versus D), giving the first clue to a possible domain swapping process.

Despite the short-time range explored in the simulations, all data indicate the destabilization of the hinge loop induced by the mutation, especially in the absence of calcium. Such a feature is in agreement with our experimental data presented on the monomer-to-dimer transition (see below).

### Kinetics and mechanism of Gly167Arg G2 dimerization

**Figure 3.**
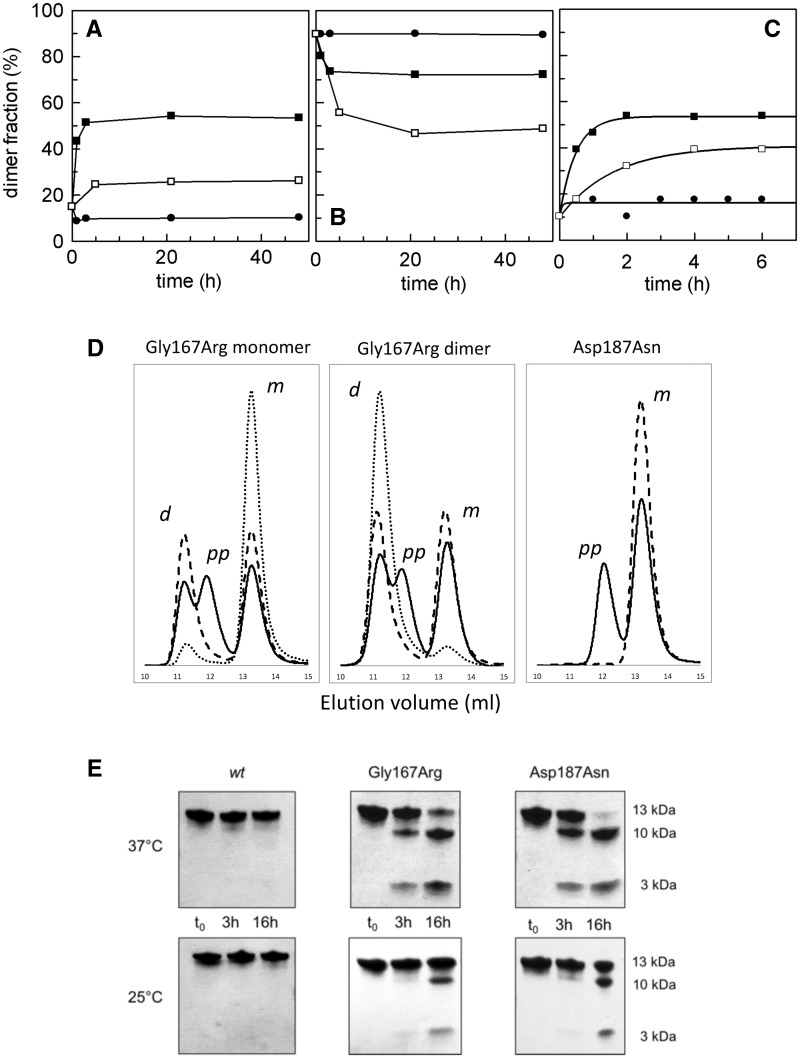
Kinetics of dimerization and susceptibility to furin proteolysis. (**A** and **B**) The kinetics of dimerization were followed by analytical gel filtration. Curves were obtained for monomers (A) or dimers (B) under three different conditions: in the presence of calcium at 4 °C (filled circle) and 25 °C (open square), in the absence of calcium at 4 °C (filled square). The fraction of dimers calculated as a fraction of total protein loaded, is plotted as a function of time (h); (**C**) as for the previous panels, the monomer to dimer transition was monitored by DLS, data were fitted with a first-order kinetics; (**D**) gel filtration profile of the Gly167Arg monomer and dimer, in comparison with the Asp187Asn variant that does not domain-swap but is susceptible to furin proteolysis. The samples were analyzed at time zero (dotted line) or after incubation at 25 °C for 16 h in the presence (continuous line) and absence (dashed line) of furin. Three elution peaks can be observed corresponding to the dimer (11.5 ml elution volume, *d*), monomer (13.5 ml, *m*) and a larger product of furin proteolysis (12 ml, *pp*). (**E** and **F**) Furin proteolysis assays were performed on Asp187Asn and Gly167Arg variants at 25 or 37 °C and visualized by SDS-PAGE.

Following the presence of the domain swapped dimer of Gly167Arg in the crystal, we carried out analytical gel filtration (GF) experiments to separate the monomeric and dimeric forms. Indeed, the two oligomeric species could be resolved by analytical GF, allowing for dimerization studies to be carried out on the solution. Two possible processes may explain the formation of the domain swapped dimer: either the dimer is formed directly during protein folding and thereafter remains stable, or the two species are in dynamic equilibrium. To test the latter hypothesis, isolated dimers and monomers were incubated under three different conditions. Time-course transitions in both directions were analyzed by GF as reported in Figure 3A and B. These analyses revealed that monomer to dimer transitions can occur. However, at low temperature (4 °C), the two isolated conformations remain well separated over a relatively long time. Both an increase in temperature (25 °C) and/or calcium depletion upon the addition of ethylenediaminetetraacetic acid (EDTA) promote the conformational transition toward a mixed population with some differences in the transition rate and in the final monomer/dimer ratio. In the tested conditions, irrespective of whether experiments commenced from monomer or dimers, the equilibrium values converge to roughly 50%. To further investigate this aspect, aliquots of Gly167Arg monomer, in a range of protein concentration between 0.2 and 2 mg/ml, were incubated for 48 h at 4 °C with 1 mM EDTA, and analyzed by GF. As expected, we found a correlation between the monomer:dimer molar ratio and the concentration of total protein (see Supplementary Material, Fig. S3). Calcium depletion seems to play a dual role in the dimerization process: (i) it destabilizes the gelsolin fold and favors protein opening facilitating the transition; and (ii) it shifts the equilibrium toward dimerization, lowering the energy associated with this conformation. These data are in accordance with the MD simulations that showed that calcium helps to maintain the stressed structure, whereas the G2 domain (and the hinge loop in particular) promptly starts to relax in the absence of calcium.

To achieve a more quantitative description of the process, we studied dimerization by dynamic light scattering (DLS) under the same conditions used in GF experiments (Fig. 3C). At 4 °C, and in the presence of calcium, we confirmed the stability of the monomer (measured hydrodynamic radius 2.0 ± 0.3 nm). In the other two conditions (i.e. at 4 °C with EDTA and at 25 °C with calcium) the conversion to dimers follows a pseudo-first-order kinetics (Fig. 3C), with calculated *k* (*t*^−1^) values of 2.1 ± 0.2/h or 0.6 ± 0.1/h, respectively. Once again, these experiments suggest that the two species are separated by a high kinetic barrier that can be lowered by removing Ca^2+^. When no significant rearrangement of the backbone is involved, protein–protein interactions are typically based on rapid association kinetics. The low rate constants we measured suggest the presence of a large conformational change supporting the domain-swapping dimerization process.

In the monomer to dimer conversion, the mutant must necessarily adopt a transition state, where the β1 strand is extruded from the core of the protein (open monomer) before being locked again into the other open monomer. To gain insight into the mechanisms of this process, we produced a truncated form of the G2 domain (Δβ1G2) that lacks N-terminal strand β1 (i.e. starting from residue Arg168). Δβ1G2 was characterized by GF and circular dichroism spectroscopy (CD) (Supplementary Material, Fig. S4). Despite the fact that Δβ1G2 cannot dimerize via domain swapping, two peaks were observed in the GF profile: a minor peak eluting at a similar elution volume (*V*_E_) to the folded monomer (*V*_E_ = 13.5 ml) and a major peak with a lower *V*_E_ of 12 ml, corresponding to a form with an increased hydrodynamic radius. CD analysis revealed that Δβ1G2 lacks any secondary structure elements and its spectrum reflects that of denatured G2 domains (Supplementary Material, Fig. S4). In conclusion, the Δβ1G2 domain behaves as a stable and soluble protein with random coil or molten globule structural characteristics. Such evidence suggests that domain swapping may be favored when the protein undergoes partial denaturation, leading to extrusion of β1. This transition state (open monomer) is not prone to aggregation or precipitation.

### Gly167Arg G2 stability and susceptibility to proteolysis

**Figure 4.**
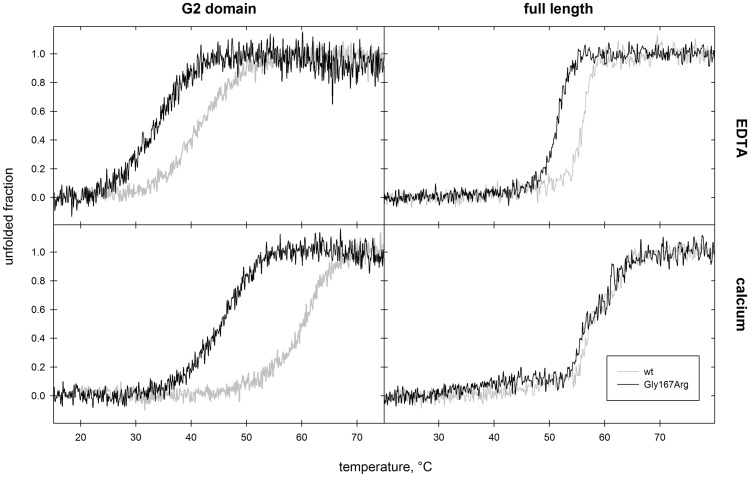
Impact of the mutation on gelsolin thermodynamic stability. Left panels: thermal denaturation of Gly167Arg (black trace) and wt (grey trace) G2, monitored by CD spectroscopy, in the absence (top) and presence (bottom) of calcium. Right panels: The same analysis was also performed on the full length proteins.

Several studies report that most monogenic disease mutations have an impact on protein folding rather than its physiological function (37). For hereditary amyloidosis, in particular, there is often a tight correlation between thermodynamic stability of the mutant protein and its propensity to aggregate. For these reasons, we analyzed the thermal stability of Gly167Arg by CD spectroscopy and compared the results with those previously obtained for other gelsolin variants (Fig. 4 and Table 3). Experiments were performed in the presence and absence of calcium to assess both functional states of the protein, and to verify the ability of the mutants to bind calcium in solution. As expected, calcium has a stabilizing effect on the G2 domain of Gly167Arg, with a Δ*T*_m_ of 12 °C. This difference is comparable with that observed for Asn184Lys, but is lower than the Δ*T*_m_ calculated for the wt, suggesting that the mutations have a lower impact on the apoprotein fold. Nevertheless, the *T*_m_ values for Gly167Arg in both conditions are at least 10 °C lower than those measured for the wt, implying that the mutation itself increases the conformational flexibility of the domain.

**Table 3.** Thermodynamic stability of gelsolin G2 and full-length

	G2		Full-length	
	+Ca	−Ca	+Ca	−Ca
**WT**	60.6^a^	41.5^a^	56.7/61.5	56.6
**Asp187Asn**	46.5^a^	46.4^a^	nd	nd
**Asn184Lys**	47.0^a^	33.8^a^	nd	nd
**Gly167Arg**	45.6	33.1	55.5/61.5	51.5

Conformational flexibility was evaluated by CD spectroscopy in the presence of either 1 mM calcium (+Ca) or 1 mM EDTA (−Ca). *T*_m_ values (± 0.3 °C) for the Gly167Arg mutant are compared with those of the wt protein and other previously characterized variants. nd, never determined values.

a Values from Ref. (29).

As previously mentioned, the first step in the amyloidogenic pathway of the Asp187Asn/Tyr and Asn184Lys mutants, is the aberrant cleavage of the protein by furin, an endogenous protease of the Golgi network, primarily involved in the maturation of exported proteins. We tested the susceptibility of the Gly167Arg mutant to furin proteolysis in conditions mimicking the Golgi environment and at two different temperatures (37 and 25 °C). Proteolysis of the G2 domain was followed by SDS-PAGE and analytical GF, with the aim to investigate which oligomeric species is most prone to degradation (Fig. 3D and E). By GF, we can easily distinguish monomers (*V*_E_ = 13.5 ml), dimers (*V*_E_=11.2 ml) and the product of furin proteolysis (*V*_E_=12 ml) (Fig. 3D). The latter has a larger *V*_E_ with respect to the undigested G2 domain, suggesting that G2 proteolysis leads to its denaturation (Figs 3D and 4A). Although it is difficult to appreciate subtle differences in our semi-quantitative assays, these results show that both forms of Gly167Arg are readily processed by furin and that the extent of their sensitivity is similar or slightly lower than that of the Asp187Asn mutant, used as a positive control. The lack of significant differences between monomers and dimers suggests that either furin processes monomers and dimers with similar efficiency or that the equilibrium between the two forms is faster than proteolysis under our experimental conditions.

### Impact of Gly167Arg mutation on the full-length protein

**Figure 5.**
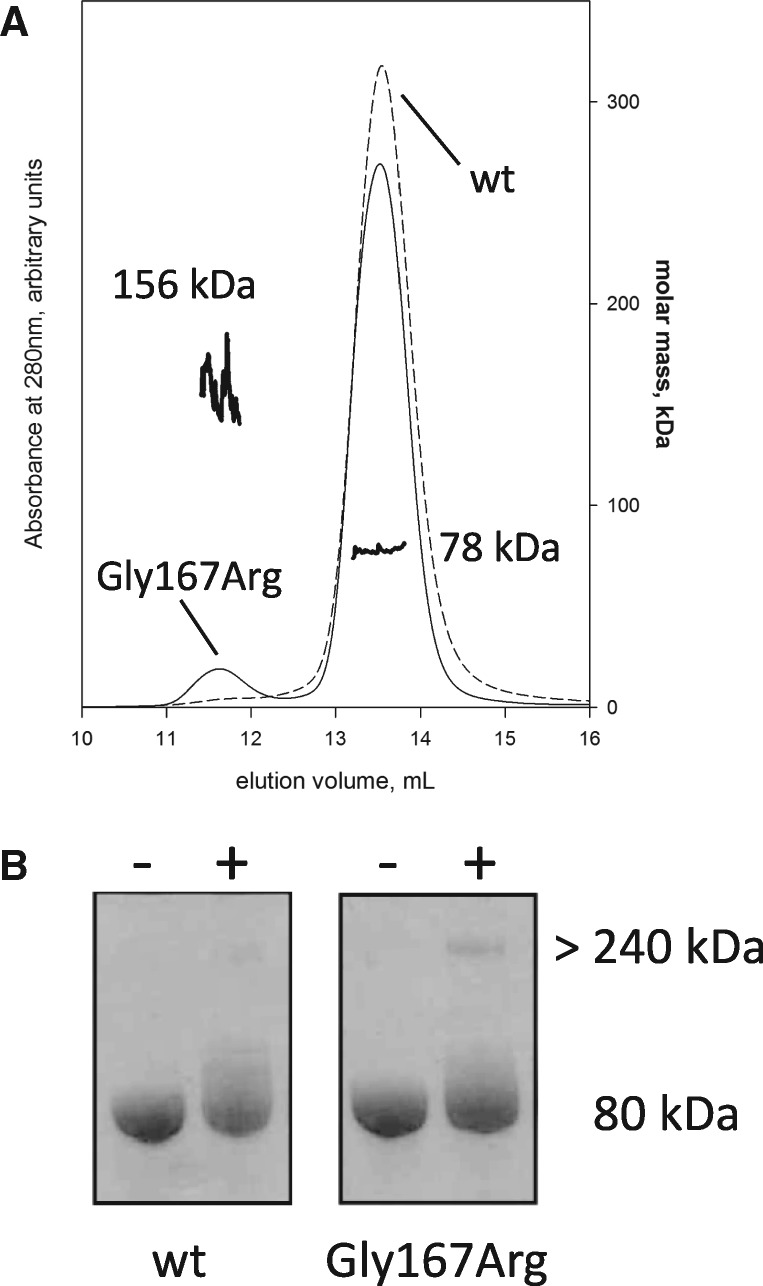
Oligomerization of the full-length mutant protein. (**A**) Left axis: analytical gel filtration elution profile of wt (continuous thin line) and Gly167Arg (dashed thin line) full length proteins in the absence of calcium. In the presence of calcium, no significant difference was visible (data not shown). Right axis: molar mass calculated on-line by MALS for the two peaks of Gly167Arg GSN (continuous thick lines). (**B**) SDS-PAGE gel of wt and mutant full length gelsolin before (−) and after (+) 30 min incubation with a crosslinking agent.

The study of the structure and of the biophysical properties of full-length gelsolin is challenged by the existence of multiple functional states and high conformational flexibility. Nevertheless, in order to validate our findings on the isolated G2 domain, full-length plasma gelsolin and the Gly167Arg pathological mutant were produced. Preliminary spectroscopic analyses ruled out a major impact of the mutation on the protein structure. CD spectra of wt and Gly167Arg gelsolin were recorded and proteins were analyzed in temperature ramp experiments under the same conditions used for the isolated G2 domains. Comparisons made between the four spectra failed to highlight any major differences, suggesting that the mutation does not significantly affect protein structure or calcium regulation (data not shown). In the presence of calcium, the temperature ramps are superimposable (with a characteristic biphasic profile) and calculated *T*_m_ values are within experimental error (Fig. 4 and Table 3). In contrast, significant differences are evident in the presence of EDTA, i.e. when the protein is in a closed conformation (inactive state). Mutant unfolding still shows high cooperativity, suggesting that the protein is properly folded but its *T*_m_ value is 6 °C lower than that measured for the wt (Fig. 4 and Table 3). GF analysis of the full-length variants highlighted differences between the wt and the mutant only for the inactive conformation (in the presence of EDTA). The Gly167Arg elution profile is characterized by two peaks: the main one corresponds to the wt protein (*V*_E_=13.5 ml) and the second to a higher molecular weight protein (*V*_E_=11.7 ml; Fig. 5A). Since no high molecular weight bands were visible by SDS-PAGE analysis (Fig. 5B), the two peaks correspond to different structural states of gelsolin. This elution profile might be the result of either the opening of the structure in a calcium-independent way (i.e. the mutation interferes with the proper regulation mechanism) or the formation of dimer/oligomers of the protein. To answer this question, we incubated wt and Gly167Arg proteins with bis(sulfosuccinimidyl)suberate (BS^3^), a crosslinking agent, in the absence of calcium and analyzed the reaction products by SDS-PAGE. As can be seen in Figure 5B, the peak eluted in GF with a *V*_E_ of 8.7 ml, corresponding to larger oligomeric assemblies of mutant gelsolin, which is covalently blocked by the crosslinker. To further investigate the nature of these oligomers we analyzed the full-length Gly167Arg by multi-angle light scattering (MALS) coupled with GF. MALS gives a reliable estimation of absolute molecular weights of macromolecules separated by GF. The light scattering measure supports the presence of a 77.5 ± 1% kDa molecule, eluting at 13.5 ml, and larger 156.6 ± 6% kDa species (*V*_E_ = 11.7 ml). These values are consistent with the monomeric and dimeric forms of gelsolin full-length, as the protein theoretical molecular weight is 83 kDa.

In conclusion, the Gly167Arg mutation promotes gelsolin dimerization in the full-length protein. Interestingly, these dimers are only observed in the absence of calcium, when the conformational stability of the protein is compromised and when the domain-swapped dimeric conformation is favored.

## Discussion

Out of the four gelsolin amyloidogenic mutations identified to date (http://www.amyloidosismutations.com; date last accessed 23 October 2017), Gly167Arg is the rarest, being found in a single family. Alternatively, it is the mutation that has eluded current diagnostic tools the most since diagnosis in the amyloidosis field is still suboptimal. Compared with other pathogenic variants, Gly167Arg mutation is also less conservative in terms of physico-chemical properties of the two amino acids. Glycine is a peculiar residue with enhanced conformational freedom, as shown by its unique Ramachandran plot. On the contrary, arginine contains a large, bulky guanidinium side-chain that carries a positive charge under all physiological pHs. Hereditary amyloidosis is classified as misfolding diseases and, indeed, mutations are responsible for conformational changes of the native state of the protein, which eventually lead to the deposition of ordered protein aggregates. Nevertheless, even in cases where the mutation drastically changes protein function and behavior, the impact on the native structure is often subtle and localized, eluding structure-based mechanistic interpretation (38). In contrast, the presence of the Gly167Arg mutation in gelsolin causes a dramatic reorganization of the polypeptide chain, forming the domain-swapped dimer. 

Domain swapping was first described in 1994 and represents a peculiar mechanism that forms protein assemblies (39). Since its discovery, accumulating evidence suggests that domain swapping-mediated oligomerization is involved in several physiological processes, exploited by nature as a regulatory mechanism (40) and to evolve novel functions (41) in a similar way to gene duplication. Domain swapping has also been associated with pathological aggregation of proteins, as in serpinopathies (42), Alzheimer’s disease (43) and the more related case of cystatin C, whose Leu68Gln mutation is responsible for another hereditary amyloidosis. Contrary to gelsolin, wt cystatin C shares the pathological variants ability to dimerize (44).

The gelsolin Gly167Arg mutation plays multiple roles in AGel. The substitution increases the conformational flexibility of the G2 domain, which becomes prone to proteolysis by furin. Aberrant cleavage by furin is the first step in the proteolytic cascade, which leads to the Finnish form of the disease. Nevertheless, mass spectrometry analysis of Gly167Arg deposits identified protein fragments different from the canonical amyloidogenic stretches (19), suggesting that the full-length protein may also aggregate via a mechanism that cannot be explained by the proteolytic pathway alone. The ability of the mutant to form domain swapped dimers implies the existence of an alternative, proteolysis-independent mechanism. Furin requires calcium for activity, therefore the proteolytic pathway may be referred to as a calcium-dependent mechanism. In contrast, the absence of calcium favors dimerization of the protein.

**Figure 6.**
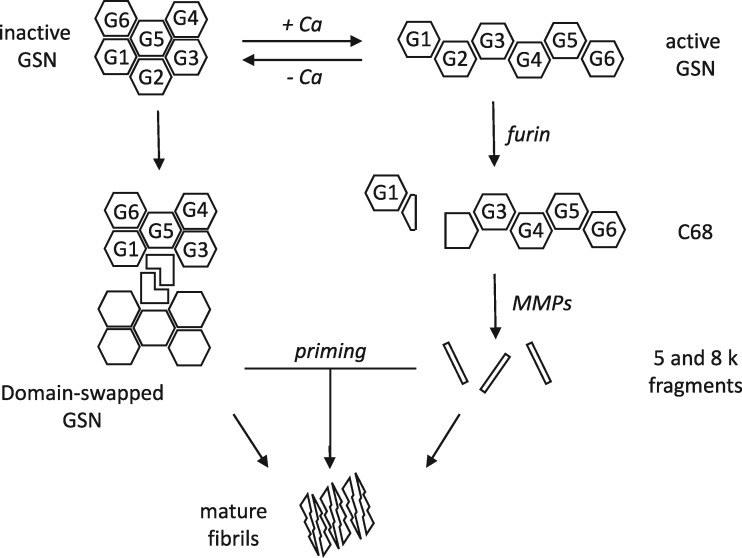
Revised mechanism of gelsolin aggregation. Schematic representation of our results integrated with the current model of AGel amyloidosis. The left side of the diagram shows the well-known proteolytic pathway, which starts with the aberrant cleavage of full length gelsolin (GSN) by furin, a calcium-dependent protease of the Golgi network. Once the C68 fragment is formed, it is exported to the extracellular space where matrix-metalloproteases (MMPs) further process the protein, producing short 5 and 8 kDa peptides. In addition to this calcium-dependent pathway, we observed dimerization of the protein in the absence of calcium, owing to the intertwining of gelsolin domain 2. These oligomers might be *per se* prone to amyloid aggregation or be primed by circulating short fragments produced by the other branch in the diagram.

In conclusion, we believe that the current AGel pathological model should be revised to integrate our findings and to take into account the coexistence of two parallel amyloidogenic mechanisms (Fig. 6): the well-known aberrant proteolytic cascade and a calcium-independent pathway that starts with protein dimerization mediated by domain swapping. There may be a potential cross-talk between the two proposed pathways, as full length oligomers may be unable to form amyloids and their aggregation may be primed by the 5 and 8 kDa fragments. Another aspect that should be investigated is the origin of the calcium-free (inactive) protein that undergoes dimerization. The plasma isoform of gelsolin, responsible for the Finnish AGel, is believed to be constitutively active, as the concentration of free calcium throughout the secretory pathway and in the plasma are sufficient for the activation of the protein. Conversely, the cytoplasmic isoform of gelsolin could be the etiological agent of this amyloidosis. Some intracellular prefibrillar aggregates of the Gly167Arg variant initially accumulated in the cytoplasm might be released in the extra cellular space upon cell death. Release of *intracellular amyloids* is a mechanism that has been recently described in some Tau- and α-synuclein-related diseases, which are now recognized as canonical amyloidosis (45,46). In the formulation of these hypotheses, we should also take into account the kidney-localized deposition of the Gly167Arg aggregates. Available clinical data are very limited and we cannot rule out the participation of other external factors in the disease, which would tag the Gly167Arg substitution as a risk factor rather than as a disease-causing mutation. Finally, the calcium-dependent activation of the Gly167Arg might be altered, although binding of the ion is not completely impaired. This would lead to the circulation of a small population of inactive gelsolin at physiological concentration of calcium, sufficient to produce the first fibrillar seeds.

Currently, the general consensus is that early oligomers and prefibrillar assemblies of amyloidogenic proteins, rather than mature fibrils, represent the most relevant species in the pathway that leads to protein deposition and toxicity (47–50). Our findings indicate that, in accordance with other protein misfolding diseases, soluble oligomeric structures may be actively involved in AGel. The identification and characterization of such structures may significantly contribute to an improved comprehension regarding the molecular mechanisms of disease, paving the way for the design of innovative pharmacological approaches.

## Materials and Methods

### Cloning and mutagenesis

Gly167Arg G2 variant (residues 151–266) and the truncated form Δβ1G2 (168–266) were produced by site-directed mutagenesis, using the *wt* construct (29) as template and the Q5^®^ Site-Directed Mutagenesis Kit (New England BioLabs). Primers were designed using the manufacturer’s software (nebasechanger.neb.com).

The nucleotide sequence encoding for mature human full-length plasma gelsolin (GSN, residues 26–782, UNIPROT P06396) was optimized according to *E. coli* codon usage. The gene was synthesized by Eurofins Genomics (Ebersberg, Germany) and cloned into a pET-28a plasmid carrying a 6xHis-tag at the N-terminus of the protein.

Recombinant plasmids (G2_Gly167Arg/pET28, Δβ1G2/pET28 and GSN/pET28, GSN_Gly167Arg/pET28) were used to transform either BL21 (DE3) or *Rosetta E. coli* cells (Invitrogen™).

### Expression and purification

The Gly167Arg G2 mutant and the Δβ1G2 variant were expressed and purified following the protocol described in (29) for the G2s construct.

GSN wt and GSN Gly167Arg were produced in BL21 and *Rosetta (DE3) pLysS??*, respectively, and the expression was induced upon addition of 1 mM IPTG for 16 h at 20 °C. Cells were harvested and resuspended in 150 ml of 20 mM sodium phosphate, 0.5 M NaCl, 10% glycerol, 20 mM imidazole, pH 7.4, supplemented with a tablet of protease inhibitor cocktail (cOmplete, EDTA-free, Roche) and 10 µg/ml of Deoxyribonuclease I from bovine pancreas.

Cells were then lysed by high pressure in a Basic Z Bench top (Constant Systems Limited, U.K.) at 25 kPSI and the crude extract was centrifuged at 38 700 RCF for 30 min and filtered through a 0.45 µm filter. The clarified soluble extract was loaded onto a 1 ml HisTrap HP column (GE Healthcare Life Sciences) and GSN variants were eluted with 20 mM sodium phosphate, 0.5 M NaCl, 10% glycerol, 0.3 M Imidazole, pH 7.4. Fractions corresponding to GSN were pooled and exchanged by desalting into 20 mM Tris–HCl pH 8, 20 mM NaCl, 10% glycerol, 1 mM EGTA, 1 mM EDTA and 1× cOmplete protease tablet.

Proteins were further purified by anion exchange chromatography on a Mono Q 5/50 GL column (GE Healthcare Life Science), eluting with a linear gradient of NaCl (20 mM to 1 M). The peaks corresponding to pure GSN were collected and stored in 20 mM HEPES, pH 7.4, 100 mM NaCl and 1 mM EGTA. All chromatographic steps were performed on a ÄKTA pure 25L (GE healthcare).

### Thermal stability

Thermal stability experiments were performed on the Gly167Arg mutant and the full-length protein using a J-810 spectropolarimeter (JASCO Corp., Tokyo, Japan) equipped with a Peltier system for temperature control. All measurements were carried out in 20 mM HEPES pH 7.4, 100 mM NaCl and either 1 mM CaCl_2_ or 1 mM EDTA at a protein concentration of 0.2 mg/ml. Temperature ramps were recorded from 10 to 95 °C for G2 and from 20 to 95 °C for GSN (slope 1 °C/min) in a cuvette with a path length of 0.1 cm and monitored at a wavelength of 218 nm. The *T*_m_ was calculated as the maximum of the first-derivative of the traces. Spectra before and after the unfolding ramp were recorded (200–260 nm).

### Analytic GF and MALS

All analytic GFs of G2 variants and Δβ1G2 were carried out using a Superdex™75 Increase 10/300 GL column (GE Healthcare Life Science). In order to follow the dimerization of the protein, 0.5 mg/ml of isolated dimer and monomer were incubated in three different conditions:

20 mM HEPES pH 7.4, 0.3 M NaCl, 1 mM CaCl_2_, 4 °C
20 mM HEPES pH 7.4, 0.3 M NaCl, 1 mM CaCl_2_, 25 °C
20 mM HEPES pH 7.4, 0.3 M NaCl, 1 mM EDTA, 4 °C

A total of 200 µl aliquots were collected at different time points and loaded into the column. The area of the single peaks was calculated as a percentage of the total area of all observed species.

Analytic GFs for full length GSN and the variant Gly167Arg were performed with 200 µl samples at a concentration of 0.5 mg/ml in 20 mM HEPES pH 7.4, 100 mM NaCl and either 1 mM EDTA or 1 mM CaCl_2_ using a Superdex™200 Increase 10/300 GL (GE Healthcare Life Science).

Molar mass of monomer and oligomer present in Gly167Arg GSN sample were characterized with a Dawn^®^ Heleos^®^ Multi Angle Light Scattering (Wyatt, Santa Barbara, CA, USA) mounted on a GF-HPLC system. Two hundred microliters of 4 mg/ml sample were separated by GF, as previously described, connected on-line with the Dawn^®^ Heleos^®^ Multi Angle Light Scattering, an Optilab^®^ T-rEX Refractive Index Detector (Wyatt) and a Waters 2487 Dual λ Absorbance Detector. Molar mass at different volumes of elution were calculated by means of Astra software (v. 5.3.4.18, Wyatt) using 0.185 as dn/dc value.

### Dynamic light scattering

A total of 1 mg/ml solutions of monomeric Gly167Arg variant were filtered through a 0.22 µm filter (Merck Millipore Ltd.) and incubated in the same three different conditions used for analytical GFs. Sixty microliters of the incubation mixtures were taken at different time intervals and the hydrodynamic radius (*R*_H_) was measured by DLS. All measurements were carried out at 4 °C in a DynaPro instrument (Protein Solutions, Charlottesville, VA, USA). The *R*_H_ measured by DLS gave a value which is proportional to the dimer:monomer ratio, therefore calibration was based on the GF data and *R*_H_ values of 2.0 ± 0.3 nm and 3.0 ± 0.3 nm used as reference for the monomer and dimer, respectively.

### Furin proteolysis assays

Furin cleavage assays were performed as reported in (29) at two different temperatures, 37 and 25 °C. Proteolysis was monitored by SDS-PAGE and by analytical GF. In the latter, protein samples (0.5 mg/ml) were analyzed as described in the previous GF section and after 16 h incubation in the furin buffer, either in the presence or the absence of furin protease.

### Crosslinking assay

In order to assess if substitution Gly167Arg promotes higher oligomeric assemblies of the full-length protein, 25 µM wt and Gly167Arg GSN were incubated with 1 mM bis(sulsosuccinimidyl)suberate (BS^3^, ThermoFisher) in 20 mM HEPES, pH 7.4, 100 mM NaCl, 1 mM EDTA at 20 °C. After 15′ incubation, the reaction was blocked by addition of 1 M Tris–HCl pH 7.5. As a negative control, the same reactions were performed in the absence of BS^3^. Products of the crosslinking reaction were analyzed by SDS-PAGE.

### Crystallization, structure solution and analysis

Vapor-diffusion crystallization experiments were carried out in a sitting drop set-up at 20 °C using an Oryx-8 nanodispenser robot (Douglas Instruments). Using 9 mg/ml G2 Gly167Arg (in 20 mM HEPES, 300 mM NaCl, pH 7.4, supplemented with 5 mM CaCl_2_) drops of 0.4 µl were prepared with different protein/precipitant ratios. In order to find optimal crystallization conditions, several commercial crystal screen solutions were tested (HTTM, Hampton Research, JCSG-plus™, Molecular Dimensions, Wizard™, Emerald BioSystems, Morpheus^®^ HT-96, Molecular Dimensions).

The best diffracting crystals grew in 0.2 M ammonium acetate, 0.1 M sodium citrate pH 5.6, 30% PEG 4000. Crystals were soaked in a cryoprotectant solution containing 20% glycerol and flash-cooled in liquid nitrogen. X-ray diffraction data were collected on the ID30B beamline (ESRF, Grenoble) at −173 °C. Data were processed and scaled using XDS (51). The Gly167Arg crystal structure was solved by Molecular replacement using Phaser (52) and the structure of the Asn184Lys variant of the G2 domain (pdb id: 5FAF) as a search model. The structure was refined with Phenix refine (53) and model building performed with COOT (54)*.* Unless otherwise stated, analysis of the structures was performed with PyMol (The PyMOL Molecular Graphics System, Version 1.8 Schrödinger, LLC) that was also used to prepare the figures. Refined model and structure factors were deposited in the protein data bank under accession code 5O2Z.

Other crystals grew in 30% PEG 3000, CHES pH 9.5, 3 mM CaCl_2_ and diffracted to 2.6 Å resolution on the ID 23-2 beamline (ESRF, Grenoble), belonging to the P2_1_ space group with cell dimensions [Å] *a* = 45.1, *b* = 39.4, *c* = 62.3 and *β* = 106°. In addition to the previously mentioned software, BUSTER (version 2.10.2, Global Phasing Ltd, Cambridge, UK) was used for refinement. The structure was partially refined to final *R*_work_/*R*_free_ values of 25.5/33.6%.

### MD simulations

The MD simulations were performed using the program NAMD2 (55). The coordinates of the wt gelsolin domain 2 (pdb-id 1KCQ) from Val158 to Ala261 were modified by introducing the Gly167Arg mutation. Using the program psfgen [part of the namd2 package (55)], H-atoms were added and partial charges assigned to every atom in the model. A virtual box was built around the protein (51.2 ×53.1×65.6 Å^3^) and filled with 4863 water molecules. The system charge was equilibrated adding NaCl 0.2 M [i.e. 22 Cl^−^ (20 without Ca^2+^) and 18 Na^+^ ions], using the vmd package (http://www.ks.uiuc.edu/Research/vmd). Harmonic constraints to fix protein atom positions during energy minimization of the solvent were applied for 0.5 ns, and the systems was then equilibrated at 310 K for an additional 0.5 ns. The two simulations were run for 80 ns, with 2 fs time steps. During the simulations, periodic boundary conditions in NPT ensemble, with Langevin temperature control (*T* = 300 K) and Langevin piston Nose–Hoover method (56), were used to maintain constant temperature and pressure, respectively. To Van der Waals interactions, a cut-off of 12 Å was applied, and switched off using a smoothing function beyond 10 Å. Eelectrostatic interactions were treated with the Particle Mesh Ewald method (57), using a grid of 100 points along each dimension of the simulation box.

## Supplementary Material

Supplementary Material is available at *HMG* online.

## Supplementary Material

Supplementary Data
