# Supplementary material for: Gelsolin pathogenic Gly167Arg mutation promotes domain-swap dimerization of the protein
Source: Hum Mol Genet. 2017 Oct 23;27(1):53–65. doi: 10.1093/hmg/ddx383 (PMC5886171; doi:10.1093/hmg/ddx383)
Supplement: Supplementary Data [file ddx383_supplementary_material.pdf]

Gelsolin pathogenic Gly167Arg mutation promotes domain-swap dimerization of the protein

Francesco Bonì[1], Mario Milani[1], Alberto Barbiroli[2], Luisa Diomede[3], Eloise Mastrangelo[1] and Matteo de Rosa[1*]

[1] CNR Istituto di Biofisica, c/o Dipartimento di Bioscienze, Università degli Studi di Milano, 20133 Milano, Italy
[2] Dipartimento di Scienze per gli Alimenti, la Nutrizione e l'Ambiente, Università degli Studi di Milano, 20133 Milano, Italy. [3] Dipartimento di Biochimica e Farmacologia Molecolare, IRCCS - Istituto di Ricerche Farmacologiche "Mario Negri", 20156 Milan, Italy. [*] To whom correspondence should be addressed teo.derosa@gmail.com, +39 02503 14900

Supplementary figures:

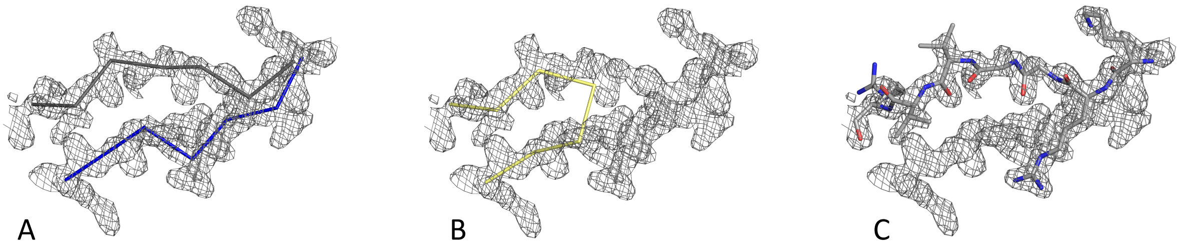

Figure S1 Electron density of the area around the hinge loop. A significant discontinuity in the 2Fo-Fc map was visible immediately after the phasing by molecular replacement hinting at an important conformational change. In the figure, electron density (contoured at 1.6 σ) was fit with the domain swap conformation of the hinge loop (**A** and **C**) or the close monomer conformation (**B**). In A and B only the Cα traces of residues 166-172 are shown while the same stretch of amino acids is represented as sticks in C.

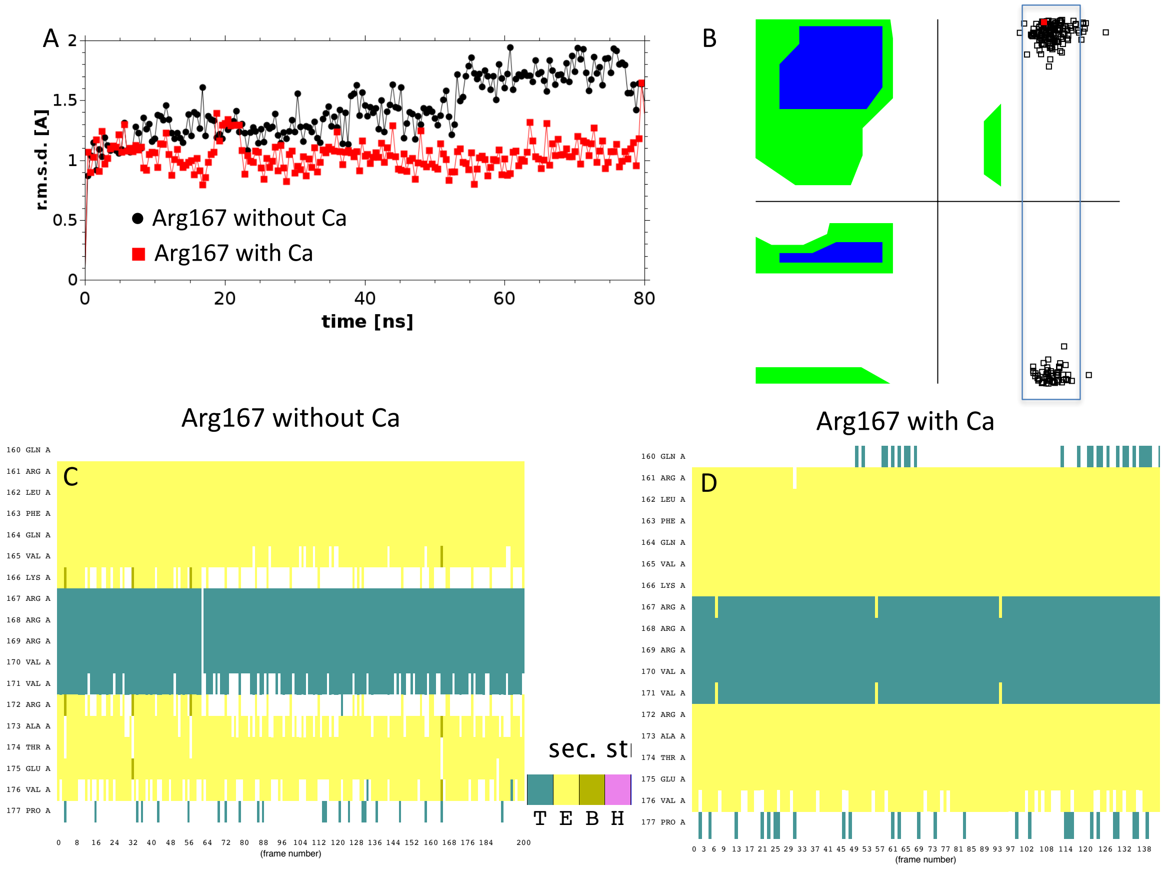

Figure S2: Impact of the mutation on the dynamics of the isolated G2 domain. A) r.m.s.d. value (Å) of the Cα atoms during the molecular dynamics simulation, computed for the Gly167Arg G2 domain in the presence (red trace) and absence (black trace) of the calcium ion. **B)** Position in the Ramachandran plot of Arg167 during the simulation in the presence of calcium. **C/D)** Secondary structure content in the surrounding of the hinge loop (residues 160-177) during the simulation in the absence (C) or presence (D) of calcium.

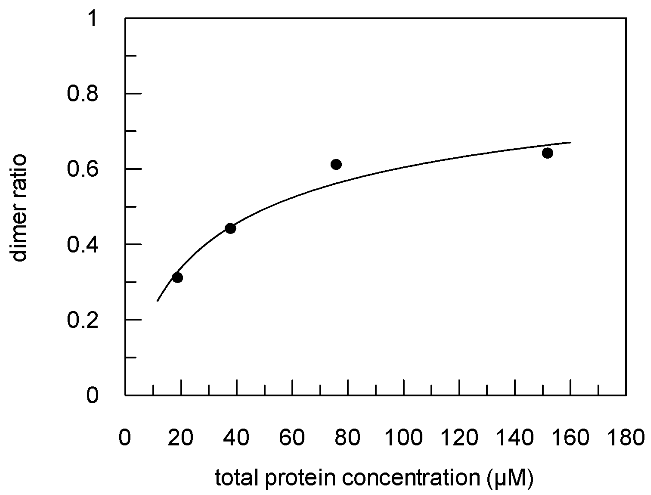

Figure S3: Concentration dependence of the dimer:monomer ratio. Aliquots of 0.2, 0.5, 1 and 2 mg/ml Gly167Arg G2 were incubated at 4°C, in the presence of 1 mM EDTA, for 48h and analysed by GF. Assuming dimerization reached an equilibrium, the molar dimeric fraction (*y*) is plotted vs total protein concentration (*x, μM)* and fit with the following equation:
$y=1-(-K_d+(K_d^2+4\ K_d\ x)^{½})/x$. From the fitting a dissociation constant (K_d) of 13±1 *μM* can be estimated.

A

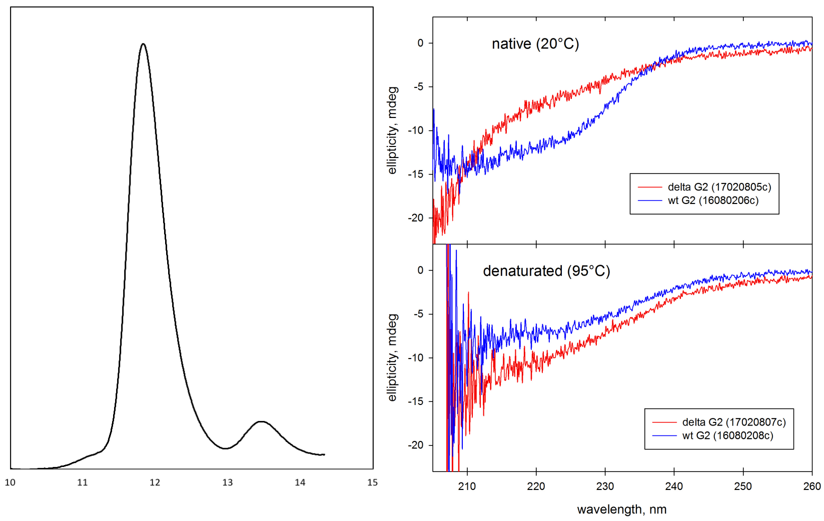

B

Figure S4: characterization of the open monomer mimic. The truncated form of G2, produced to study the open monomer conformation, transition state of the conformational change, was analysed by **A)** analytical gel filtration and **B)** circular dichroism spectroscopy, spectra were recorded at both 20 °C and after an incubation at 95 °C, to visualize the secondary structure content in native and denaturant condition, respectively. For comparison spectra of the wt G2 are reported in the same conditions.
